# Hypoglossal Nerve Stimulation Therapy for Pediatric Obstructive Sleep Apnea: A Meta-Analysis

**DOI:** 10.3390/biomedicines14040770

**Published:** 2026-03-28

**Authors:** Ji Ho Choi, Soo Kyoung Park, Jae Hoon Cho, Ji Eun Moon, Seok Hyun Cho

**Affiliations:** 1 Department of Otorhinolaryngology-Head and Neck Surgery, Soonchunhyang University Bucheon Hospital, Soonchunhyang University College of Medicine, 170, Jomaru-ro, Bucheon 14584, Republic of Korea; 2 Department of Otorhinolaryngology-Head and Neck Surgery, Chungnam National University Sejong Hospital, College of Medicine, Chungnam National University, Daejeon 34134, Republic of Korea; pacsoo2@cnuh.co.kr; 3 Department of Otorhinolaryngology-Head and Neck Surgery, Konkuk University School of Medicine, 120-1, Neungdong-ro, Gwangjin-gu, Seoul 05030, Republic of Korea; jaehoon@kuh.ac.kr; 4 Graduate School, Chungwoon University, Incheon 22100, Republic of Korea; 5 Department of Otolaryngology-Head and Neck Surgery, Hanyang University College of Medicine, 222-1, Wangsimni-ro, Seongdong-gu, Seoul 04763, Republic of Korea; shcho@hanyang.ac.kr

**Keywords:** obstructive sleep apnea, child, hypoglossal nerve stimulation, apnea-hypopnea index

## Abstract

**Background/Objectives:** This study evaluates the efficacy of hypoglossal nerve stimulation as an alternative intervention for pediatric patients with obstructive sleep apnea (OSA) unresponsive to standard therapies and examines the uniformity of therapeutic outcomes across different patient cohorts. **Methods:** An extensive systematic search was performed across four principal databases (PubMed, EMBASE, Cochrane Library, and Web of Science) utilizing keywords associated with pediatric OSA and hypoglossal nerve stimulation, encompassing studies up to July 2025 that provided objective polysomnographic metrics (e.g., apnea-hypopnea index [AHI] values) to enable the quantitative assessment of pre- and post-intervention effects in children. The primary outcome measured was the ratio of means (ROM), determined from pre–post data in single-group studies, with summary estimates obtained using the fixed-effects model. **Results:** The systematic review included nine eligible studies with a total of 140 pediatric subjects, the majority of whom were adolescents with Down syndrome. AHI meta-analysis outcomes indicated a marked improvement in OSA severity, yielding an overall ROM of 0.57 [95% confidence interval: 0.49–0.65]. The therapeutic benefit demonstrated a high degree of uniformity across cohorts, as indicated by minimal statistical heterogeneity (I^2^ = 16%, *p* = 0.30). Funnel plot assessment showed no statistically significant evidence of systematic publication bias. **Conclusions:** Current evidence suggests that hypoglossal nerve stimulation therapy is a safe, effective, and valuable alternative for pediatric OSA patients who do not respond to conventional therapies.

## 1. Introduction

Obstructive sleep apnea (OSA) in children is defined by the repetitive partial or complete collapse of the upper airway during sleep, resulting in interruptions in normal breathing and sleep architecture [1]. Affecting about 1.8% to 6.4% of the general pediatric population, OSA may present at any age from infancy to adolescence [2]. If left untreated, pediatric OSA can have substantial and enduring health impacts, such as growth impairment (e.g., failure to thrive), behavioral disturbances (e.g., attention deficit, hyperactivity), learning difficulties, and cardiovascular complications (e.g., hypertension) [3,4,5]. Because of the breadth and severity of these outcomes, early detection and intervention for pediatric OSA are critical [6]. Adenotonsillar hypertrophy is recognized as the leading cause of OSA in children, positioning adenotonsillectomy as the standard and most effective initial treatment [7]. Comprehensive management strategies, including weight control and orthodontic interventions, are frequently employed to optimize treatment outcomes [8,9].

Despite these approaches, a substantial subset of the pediatric OSA population exhibits complex anatomical and neuromuscular abnormalities that reduce the success of conventional therapies. Within this subset, children with Down syndrome, a congenital chromosomal disorder (trisomy 21), demonstrate a significantly increased OSA prevalence, frequently surpassing 75%, a marked contrast to rates seen in the broader pediatric population [10,11]. This substantial risk elevation is attributed to a combination of structural and neuromuscular features such as pharyngeal muscle hypotonia, midface hypoplasia, macroglossia, and adenotonsillar hypertrophy [12]. Owing to these anatomical and physiological susceptibilities, children with Down syndrome routinely experience severe sleep disturbances and OSA, which profoundly impact their daytime neurocognitive performance, learning capacity, and overall quality of life, while also increasing their vulnerability to cardiovascular complications, particularly because of the high hypertension and early-onset cardiac disease comorbidities in this group [13]. The American Academy of Pediatrics strongly advises that children with Down syndrome undergo polysomnography screening before 4 years of age, as caregivers frequently do not identify the symptoms of sleep-disordered breathing [14]. Notably, persistent OSA commonly occurs in children with Down syndrome following adenotonsillectomy, and there is often poor adherence to positive airway pressure (PAP) therapy, underscoring the urgent requirement for alternative, effective therapeutic strategies in this vulnerable population [15,16].

Hypoglossal nerve stimulation therapy has emerged as an innovative treatment modality aimed at alleviating upper airway obstruction during sleep [17]. This therapy operates by electrically activating the hypoglossal nerve, thereby restoring tongue muscle tone and promoting airway patency. The system consists of an implanted pulse generator, a sensing lead that tracks respiratory patterns in the chest, and a stimulating electrode applied to the hypoglossal nerve, which automatically provides stimulation in concert with the patient’s breathing cycle. Initial approval for hypoglossal nerve stimulation was granted by the U.S. Food and Drug Administration for adults with OSA in 2014. Its indication was expanded in March 2023 to include pediatric OSA patients aged 13 years and older with Down syndrome, identifying it as a promising alternative therapy [18].

Despite growing utilization, an important knowledge gap persists. Although hypoglossal nerve stimulation has been introduced for complex pediatric populations in whom adenotonsillectomy is ineffective, or PAP is not tolerated, robust quantitative assessments of its overall efficacy in pediatric OSA are notably limited. Thus, the aim of this study was to conduct a meta-analysis to assess the therapeutic effectiveness of hypoglossal nerve stimulation in pediatric OSA, providing comprehensive and evidence-based insight into its potential role in managing this challenging disorder.

## 2. Materials and Methods

### 2.1. Study Design

This research was structured as a systematic review and meta-analysis to determine the efficacy of hypoglossal nerve stimulation therapy in pediatric OSA patients. The analysis specifically targeted clinical studies that evaluated objective respiratory endpoints from polysomnography (such as apnea-hypopnea index [AHI]) values both prior to and following hypoglossal nerve stimulation therapy. The present systematic review adhered to the standards set forth in the PRISMA 2020 statement (http://www.prismastatement.org/PRISMAStatement/CitingAndUsingPRISMA [accessed on 3 February 2026]). The study protocol was registered with PROSPERO (registration number: CRD420261301233).

### 2.2. Search Strategy and Study Selection Criteria

An exhaustive literature search was performed across four principal electronic databases: PubMed (Medline), EMBASE, Cochrane Library, and Web of Science. We also manually screened the reference lists of relevant articles identified to ensure comprehensive coverage. Only studies published in English were included. The following search terms were applied: “pediatric,” “children,” “obstructive sleep apnea,” and “hypoglossal nerve stimulation.”

Studies were included in the meta-analysis if they satisfied the following criteria: (1) the study population comprised pediatric patients, (2) patients had a formal diagnosis of OSA, (3) the study evaluated the therapeutic efficacy of hypoglossal nerve stimulation, and (4) the study reported objective respiratory disturbance indices from polysomnography (e.g., AHI), enabling quantitative data extraction. Studies were excluded if they matched any of the following: (1) exclusively included adult patients, (2) enrolled subjects with isolated snoring or if the description of the patient population was ambiguous or inadequate, (3) omitted necessary data for meta-analysis (such as means, standard deviations, or pre/post-intervention values), and (4) published in languages other than English.

### 2.3. Risk of Bias Assessment

We evaluated the risk of bias for all included studies using the Risk Of Bias In Non-randomized Studies-of Interventions (ROBINS-I) tool to objectively assess the quality of the studies included in this meta-analysis. Given that most of the included studies were uncontrolled case series, we performed a detailed assessment across key domains, including bias due to confounding, selection of participants, measurement of outcomes, and attrition. The risk of bias for each study was categorized into four levels: low, moderate, serious, or critical.

### 2.4. Data Extraction and Effect Measure

Following the initial screening of titles and abstracts, full texts of selected articles were scrutinized for eligibility, and data extraction was undertaken from the final eligible publications. Two independent reviewers performed the literature search and extracted data. Discrepancies were resolved through consensus, with arbitration by a third reviewer when necessary.

The certainty of evidence for each outcome was evaluated using the Grading of Recommendations Assessment, Development and Evaluation (GRADE) approach. We assessed five specific domains: risk of bias, inconsistency, indirectness, imprecision, and publication bias. Certainty was categorized into four levels: high, moderate, low, and very low.

For AHI, designated as the primary outcome of this study, the ratio of means (ROM) was used as the effect size metric. Selecting the ROM enabled the quantification of the relative changes in average effect sizes from pre- to post-treatment. The use of a consistent AHI scale across all included studies rendered any additional standardization prior to ROM calculation unnecessary.

### 2.5. Statistical Analysis

Meta-analysis was performed using pre–post data collected from single-group studies. The ROM was used as the effect size measure to compare outcomes before and after treatment. Summary estimations were generated using fixed-effects and random-effects models. Between-study heterogeneity was assessed using both the I^2^ statistic and the between-study variance estimate (τ^2^). Heterogeneity was interpreted as low for an I^2^ value below 25%, moderate around 50%, and high for 75% or greater. Egger’s regression test was applied to identify potential publication bias. The final pooled ROM effect size and corresponding 95% confidence interval (CI) are presented. The percentage change in the measure after intervention relative to baseline was determined by (estimated ROM − 1) × 100 (%). Results with a *p*-value of less than 0.05 were considered statistically significant. All analyses were executed using Rex version 3.6.3 (RexSoft Inc., Seoul, Republic of Korea; https://rexsoft.org).

## 3. Results

**Figure 1:**
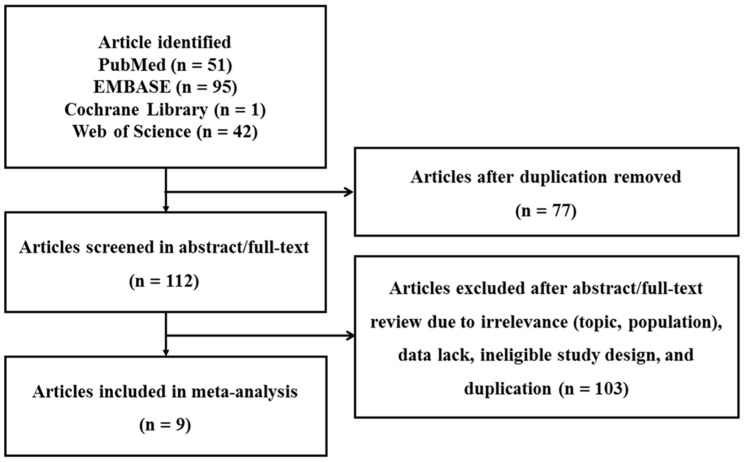
Diagram illustrating the study selection process.

The systematic literature search across the four major databases, PubMed (n = 51), EMBASE (n = 95), Cochrane Library (n = 1), and Web of Science (n = 42), initially identified a total of 189 records. After duplicate records were removed during the initial screening, 77 redundant entries were excluded, yielding 112 unique articles for further evaluation. These articles then underwent both abstract and full-text reviews. Of these, 103 articles were excluded for reasons such as irrelevance to the research focus or study population, insufficient statistical data required for meta-analysis, inappropriate study design (case reports), or duplication undetected in the first stage. Ultimately, nine articles met all predetermined inclusion criteria and were included in the final quantitative synthesis (meta-analysis) [19,20,21,22,23,24,25,26,27]. Figure 1 provides a detailed overview of the study selection process.

Table 1 summarizes the characteristics of the nine studies assessing the efficacy of hypoglossal nerve stimulation therapy in pediatric OSA, with a combined total of 140 subjects. Baseline demographics indicated that the mean participant age was largely concentrated between 14 and 16 years. Aggregate sex distribution showed a predominance of males (male:female = 91:49). Baseline anthropometric data indicated a relatively elevated mean body mass index (BMI) in the pediatric population studied, suggesting an overweight or obese cohort by standard classifications. The methodological quality among the studies varied, with most constituting Level IV evidence (case series), complemented by two prospective cohort studies and one retrospective cohort study.

The risk-of-bias assessment for the nine included studies was conducted using the ROBINS-I tool. Overall, seven studies were judged to have a serious risk of bias, while two prospective cohort studies (Yu et al. [23] and Larrow et al. [27]) were rated as having a moderate risk. The serious risk of bias observed in most of the studies was primarily driven by the confounding and participant selection domains, which are inherent limitations of the uncontrolled case series design. In contrast, the risk of bias related to the classification of interventions and outcome measurement was consistently low across all studies, largely due to the use of objective polysomnography for AHI measurement. A moderate risk of bias due to missing data was noted in three studies (Caloway et al. [21], Marcus et al. [24], and Mecham et al. [25]), while all other domains remained at a low risk of bias.

Based on the GRADE assessment, the overall certainty of the evidence regarding the efficacy of hypoglossal nerve stimulation in the pediatric population was judged to be very low. Although the reduction in AHI values was consistent across studies, the evidence was downgraded primarily due to the study design (uncontrolled case series), which inherently carries a high risk of bias and concerns regarding indirectness. Furthermore, the small sample sizes in several studies contributed to imprecision, preventing a higher certainty rating.

Detailed outcomes for each study concerning preoperative and postoperative AHI are summarized in Table 2. The range of preoperative AHI values was broad, extending from 23.5 ± 9.7 events/h to 79.3 ± 48.1 events/h, which highlights notable variability in initial disease severity. In the postoperative phase, all studies reported marked declines in AHI, with mean results ranging from 3.7 ± 1.9 events/h to 13.1 ± 9.8 events/h. Additionally, for studies that provided data, post-treatment OSA-18 survey scores showed significant decreases, consistent with improved disease-specific quality of life following hypoglossal nerve stimulation.

**Figure 2:**
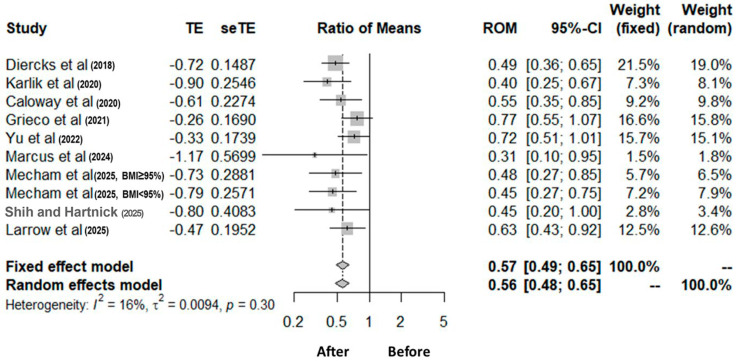
Forest plot depicting the effect of hypoglossal nerve stimulation therapy in pediatric obstructive sleep apnea. The analysis shows a statistically significant reduction in the mean apnea-hypopnea index after hypoglossal nerve stimulation compared to baseline values before intervention. TE, treatment effect; seTE, standard error of treatment effect; ROM, ratio of means; CI, confidence interval [19,20,21,22,23,24,25,26,27].

A meta-analysis of AHI decreases was conducted by aggregating results through the ROM, defined as the ratio of postoperative AHI to preoperative AHI (Figure 2). The pooled findings revealed a statistically significant reduction in OSA severity post-hypoglossal nerve stimulation, with a summary ROM of 0.57 (95% CI: 0.49–0.65) under the fixed effects model, which was consistent with the random-effects model results (ROM 0.56; 95% CI: 0.47–0.66). Heterogeneity among the included studies was minimal (I^2^ = 16%, *p* = 0.30), indicating a high degree of consistency in treatment effects. Each study consistently reported reductions in AHI after hypoglossal nerve stimulation, with ROM values from 0.31 to 0.77, which demonstrates variable yet generally favorable outcomes.

**Figure 3:**
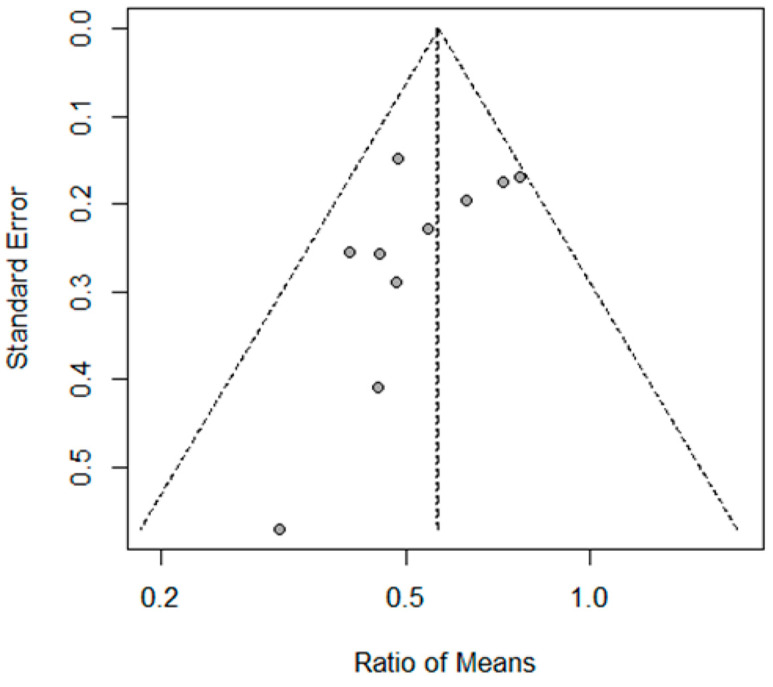
Funnel plot assessing the effect of hypoglossal nerve stimulation therapy in pediatric obstructive sleep apnea. No evidence of publication bias was detected (Egger’s test *p*-value = 0.105).

The possibility of publication bias was visually evaluated using a funnel plot, which presents the standard error versus the ROM (Figure 3). While the data points are distributed around the pooled effect size, the interpretation of this plot is limited by the small number of included studies and the predominance of small case series. Although the visual pattern does not provide definitive evidence of a substantial risk of publication bias, these results should be interpreted with caution. Collectively, the findings suggest that hypoglossal nerve stimulation yields a significant reduction in the severity of pediatric OSA, with consistent therapeutic outcomes across various study populations. However, given the statistical limitations of the funnel plot in this context, the absence of observed asymmetry does not entirely rule out the potential for publication bias.

## 4. Discussion

This meta-analysis presents a detailed, quantitative assessment of the therapeutic effectiveness of hypoglossal nerve stimulation therapy in pediatric OSA patients, including those with complex medical comorbidities such as Down syndrome who have not responded to standard interventions such as adenotonsillectomy or PAP. After an extensive literature review and strict study selection, nine studies met the inclusion criteria for pooled analysis, suggesting that hypoglossal nerve stimulation may reduce OSA severity in carefully selected pediatric patients, although the current evidence remains limited.

In this study, hypoglossal nerve stimulation therapy yielded a significant improvement in OSA severity, as evidenced by the pooled summary ROM of 0.57 (95% CI, 0.49–0.65) under the fixed-effects model and 0.56 (95% CI, 0.47–0.66) under the random-effects model.

The pooled ROM of 0.56–0.57 indicates a significant reduction of 43–44% in the AHI after hypoglossal nerve stimulation therapy [19,20,21,22,23,24,25,26,27]. These findings suggest that hypoglossal nerve stimulation may provide clinically meaningful improvement in OSA severity in selected pediatric populations. The narrow range of ROM values observed among individual studies (0.31–0.77) highlights the consistency of treatment efficacy. In addition, the low level of statistical heterogeneity detected (I^2^ = 16%, *p* = 0.30) provides further evidence that this therapeutic modality produces consistent treatment effects across diverse patient cohorts. Further, the assessment of publication bias using the funnel plot showed that the individual studies were distributed around the summary effect size. However, the interpretation of this distribution is limited by the small number of included studies (k < 10). While no definitive evidence of significant asymmetry was observed, this result should be interpreted with caution, as the statistical power to detect publication bias is reduced in small samples. Diercks et al. [28] described the first pediatric implantation of a hypoglossal nerve stimulator in an adolescent with Down syndrome and refractory severe OSA (baseline AHI: 48.5 events/h) who had failed PAP therapy and had an established tracheotomy. Treatment with hypoglossal nerve stimulation was well-tolerated and highly efficacious, leading to a substantial reduction in overall AHI to 3.4 events/h. Importantly, this successful intervention enabled permanent decannulation of the tracheotomy 5 months after implantation, demonstrating the potential of hypoglossal nerve stimulation therapy to prevent the need for tracheotomy in selected pediatric patients. Mecham et al. [25] performed a retrospective cohort study evaluating the outcomes of hypoglossal nerve stimulation therapy in 20 pediatric patients with Down syndrome, focusing on the relationship between BMI and AHI reduction. The analysis showed no significant correlation between the BMI percentile and AHI reduction (r = 0.06, *p* = 0.8), and no significant differences were observed in preoperative AHI, postoperative AHI, or AHI reduction between obese and non-obese groups. Both cohorts demonstrated favorable responses, with AHI reductions of 83.8% in obese patients and 81.9% in non-obese patients, indicating that the BMI does not compromise the effectiveness of hypoglossal nerve stimulation in this pediatric population. Larrow et al. [27] conducted a comprehensive assessment of the long-term outcomes of hypoglossal nerve stimulation in 33 adolescents with Down syndrome and persistent severe OSA, with an average follow-up period of 4 years after implantation [27]. The results indicated substantial and consistent long-term effectiveness: the mean AHI reduction from baseline was 15.7 events/h at long-term follow-up (timepoint 3, 4.0 ± 1.9 years after hypoglossal nerve stimulation), corresponding to a mean percentage decrease in AHI of 59.6%. Importantly, the long-term response rate, defined as a 50% reduction in AHI, was 87.9%, indicating that patients not only preserve initial improvements but may continue to show further enhancement in polysomnographic parameters over time.

Although limited in number, the studies included in this meta-analysis that evaluated both AHI and OSA-18 scores consistently demonstrated a significant reduction in OSA-18 scores following hypoglossal nerve stimulation therapy. This finding indicates that the impact of hypoglossal nerve stimulation extends beyond respiratory metrics to encompass substantial improvements in disease-specific quality of life. Given that OSA in children can markedly impair cognitive function, behavioral development, and cardiovascular health, the improvement in quality of life highlights the significant clinical importance of this intervention. Caloway et al. [21] conducted a case series investigating the safety and efficacy of hypoglossal nerve stimulation in 20 non-obese children and adolescents with Down syndrome and treatment-resistant severe OSA. The study demonstrated the therapy’s safety, with a high median adherence rate of 9.21 h/night, and showed notable objective efficacy, with a median percent reduction in AHI of 85% at 2 months after implantation. Additionally, hypoglossal nerve stimulation resulted in better subjective outcomes, illustrated by a median change in the OSA-18 score of 1.15, representing a moderate yet clinically important improvement in quality of life. Yu et al. [23] conducted the largest prospective study to date on hypoglossal nerve stimulation in the pediatric population, assessing the safety and efficacy of the intervention in 42 adolescents with Down syndrome and persistent severe OSA. This investigation found substantial objective and subjective benefits after 12 months, including a mean AHI reduction of 12.9 events/h with a 65.9% response rate, along with a pronounced improvement in quality of life, reflected by a decrease in the OSA-18 survey score from a baseline of 3.5 to 1.7 (mean change: −1.8). This study, which had a high rate of adherence (95.2% nightly use > 4 h), established that hypoglossal nerve stimulation therapy is a safe, efficacious, and well-tolerated option with a low rate of complications (11.9% temporary discomfort) for this difficult pediatric population.

Safety is a particularly important consideration in pediatric patients undergoing the implantation of an upper airway stimulation device. Across the included studies, reported adverse events were generally limited and often transient, including postoperative discomfort or temporary tongue weakness. However, safety reporting was inconsistent across studies, and the detailed pooled analysis of device-related complications such as infection, revision surgery, or explanation was not feasible [29]. Therefore, the current evidence supports the cautious interpretation of safety outcomes, and larger studies with standardized adverse-event reporting are needed.

From a clinical perspective, these findings may be most applicable to highly selected adolescents with Down syndrome and persistent OSA after failure of conventional treatment. The pediatric evidence base is smaller and more heterogeneous than the adult hypoglossal nerve stimulation literature, and its interpretation is influenced by syndromic craniofacial and neuromuscular characteristics [30]. In children with Down syndrome, factors such as midface hypoplasia, relative macroglossia, glossoptosis, generalized hypotonia, obesity, and multilevel upper airway obstruction may influence both candidacy and therapeutic response [31]. Accordingly, careful multidisciplinary selection remains essential, incorporating polysomnography, airway evaluation, comorbidity assessment, and individualized treatment goals [29,32]. Although early and intermediate outcomes appear encouraging, long-term durability and broader functional outcomes in pediatric populations remain incompletely defined, and further prospective studies are needed [27].

Future studies should also consider how emerging artificial intelligence and machine learning approaches may complement diagnostic stratification and patient selection in pediatric upper airway obstructive disorders, although their clinical utility in pediatric OSA remains to be more clearly defined [33].This meta-analysis presents several limitations. The primary limitation arises from the generally low quality of evidence among the included studies. Most of the research consisted of small-scale case series or retrospective, single-center analyses, rendering the pooled results vulnerable to multiple sources of selection and reporting bias. Second, considerable heterogeneity in follow-up intervals, combined with short observation periods, hampers the robust evaluation of the long-term efficacy and safety of hypoglossal nerve stimulation, particularly regarding device durability and long-term side effects in pediatric populations. Third, the studies did not adequately control for important patient-related variables. Thus, interpreting these results should be approached cautiously, as confounding factors such as variable patient age (ranging from 13 to 18 years and occasionally including young adults), the degree of obesity, and upper airway anatomical features (e.g., tonsil size or extent of tongue base collapse) complicate the analysis. Future investigations should focus on prospective, multicenter designs with larger cohorts and prolonged follow-up to enhance the strength of evidence. Such studies will be critical for establishing definitive criteria for patient selection and for verifying the long-term safety and reliability of hypoglossal nerve stimulation among children and adolescents with OSA.

This systematic review was conducted in accordance with the PRISMA 2020 statement [34], and the detailed search strategies and screening processes are provided in the Supplementary Materials.

## 5. Conclusions

Current evidence suggests that hypoglossal nerve stimulation may be a beneficial treatment option for carefully selected pediatric patients with persistent OSA, particularly adolescents with Down syndrome who have not responded to conventional therapies; however, the certainty of evidence remains limited. This observation is supported by our pooled analysis, demonstrating a significant and clinically relevant decrease in AHI, with consistency across studies and low heterogeneity, even in the presence of variable BMI values and disease severity at baseline. In addition, improvements in OSA-18 quality-of-life scores, albeit only documented in some studies, suggest that hypoglossal nerve stimulation may benefit both objective respiratory function and the broader aspects of daily life and well-being. From a clinical perspective, these findings emphasize the potential clinical value of hypoglossal nerve stimulation as a practical and durable surgical alternative for complex pediatric OSA, notably in cases resistant to first-line therapies. Integrating hypoglossal nerve stimulation into multidisciplinary treatment frameworks could address significant needs in this patient group, offering persistent symptom relief and enhanced quality of life over extended follow-up. Consequently, hypoglossal nerve stimulation therapy signifies an important advancement in pediatric sleep medicine, broadening available treatment options for this difficult-to-manage disorder.

## Figures and Tables

**Table 1:** Summary of studies evaluating the efficacy of hypoglossal nerve stimulation therapy in pediatric obstructive sleep apnea.

Author, Year	Number of Cases	Age (Years)	Sex (M:F)	BMI (kg/m^2^)	AHI (events/h)	Primary Outcome Variables	Level of Evidence (Study Design)
Diercks et al., 2018 [19]	6	14.8 ± 2.3	4:2	24.1 ± 3.7	26.4 ± 12.4	OSA-18 questionnaire scores, AHI (events/h)	Level IV (case series)
Karlik et al., 2020 [20]	3	14.0 ± 4.6	1:2	NR	29.7 ± 8.6	Obstructive AHI (events/h)	Level IV (case series)
Caloway et al., 2020 [21]	20	16 (median)	13:7	24.1 (median)	26.7 ± 10.1	OSA-18 questionnaire scores, AHI (events/h)	Level IV (case series)
Grieco et al., 2021 [22]	9	15.2 ± 3.4	5:4	25.4 ± 4.6	24.1 ± 12.3	Neurocognitive and behavioral assessments, AHI (events/h)	Level IV (case series)
Yu et al., 2022 [23]	42	15.1 ± 3.0	28:14	NR	23.5 ± 9.7	OSA-18 survey, Epworth Sleepiness Scale, polysomnographic measurements such as AHI (events/h)	Level II (prospective cohort study)
Marcus et al., 2024 [24]	3	15.3 ± 5.9	0:3	NR	79.3 ± 48.1	AHI (events/h)	Level IV (case series)
Mecham et al., 2025 [25] (BMI ≥ 95%)	8	13.9 ± 5.4	16:4	BMI ≥ 95%	26.3 ± 10.9	AHI (events/h)	Level III (retrospective cohort study)
Mecham et al., 2025 [25] (BMI < 95%)	12	14.4 ± 6.1		BMI < 95%	25.4 ± 15.0		
Shih and Hartnick, 2025 [26]	4	14.5 (median)	4:0	25 < BMI < 50	29.0 ± 12.6	Obstructive AHI (events/h)	Level IV (case series)
Larrow et al., 2025 [27]	33	NR	20:13	NR	23.8 ± 10.0	Polysomnographic variables including AHI (events/h)	Level II (prospective cohort study)

BMI, body mass index; AHI, apnea-hypopnea index; OSA, obstructive sleep apnea; M, male; F, female; NR, not reported.

**Table 2:** Outcomes of hypoglossal nerve stimulation therapy on respiratory disturbance metrics in pediatric obstructive sleep apnea.

Author, Year	Number of Cases	Preoperative AHI (events/h)	Postoperative AHI (events/h)	Preoperative OSA-18 Assessment (Survey Score)	Postoperative OSA-18 Assessment (Survey Score)
Diercks et al., 2018 [19]	6	26.4 ± 12.4	5.0 ± 1.6	NR	NR
Karlik et al., 2020 [20]	3	29.7 ± 8.6	3.7 ± 1.9	NR	NR
Caloway et al., 2020 [21]	20	26.7 ± 10.1	6.6 ± 7.3	3.5 ± 1.3	2.5 ± 1.2
Grieco et al., 2021 [22]	9	24.1 ± 12.3	13.1 ± 9.8	NR	NR
Yu et al., 2022 [23]	42	23.5 ± 9.7	11.0 ± 13.4	3.5 ± 1.2	1.7 ± 0.7
Marcus et al., 2024 [24]	3	79.3 ± 48.1	5.4 ± 5.2	NR	NR
Mecham et al., 2025 [25] (BMI ≥ 95%)	8	26.3 ± 10.9	4.9 ± 4.6	NR	NR
Mecham et al., 2025 [25] (BMI < 95%)	12	25.4 ± 15.0	4.1 ± 4.2	NR	NR
Shih and Hartnick, 2025 [26]	4	29.0 ± 12.6	4.6 ± 2.3	NR	NR
Larrow et al., 2025 [27]	33	23.8 ± 10.0	8.1 ± 8.4	NR	NR

AHI, apnea-hypopnea index; OSA, obstructive sleep apnea; NR, not reported.

## Data Availability

The datasets used and/or analyzed during the current study are available from the corresponding author upon request.

## Supplementary Materials

The following supporting information can be downloaded at: https://www.mdpi.com/article/10.3390/biomedicines14040770/s1, Reference [34] are cited in the supplementary materials.

## Abbreviations

The following abbreviations are used in this manuscript:

OSA	Obstructive sleep apnea
PAP	Positive airway pressure
AHI	Apnea-hypopnea index
ROM	Ratio of means
BMI	Body mass index
CI	Confidence interval
